# Enhancing Urban and Suburban Green Spaces: Dendrological Diversity and Climate Adaptation

**DOI:** 10.1155/tswj/4061845

**Published:** 2026-03-26

**Authors:** Zhirayr Hmayak Vardanyan, Janna Andranik Akopian, Maxim Viktorovich Larionov, Gayane Manuk Gatrchyan, Nelli Norik Muradyan, Sergey Artur Ktrakyan, Hovik Yakhshibek Sayadyan, Manik Meruzhan Grigoryan

**Affiliations:** ^1^ Department of Plant Introduction, Tree-Bush Plant Viability Research Laboratory, A. Takhtajan Institute of Botany of the National Academy of Sciences of the Republic of Armenia (NASRA), Yerevan, Armenia; ^2^ Department of Scientific Projects, Federal State Budgetary Educational Institution of Higher Education Russian Biotechnological University (ROSBIOTECH University), Moscow, Russia

**Keywords:** climate change, dendrological composition, ecological adaptation, ecological and botanical traits, ornamental value, species and varieties, species dynamics, trees and shrubs, urban green spaces

## Abstract

This study presents the first integrated, multicity assessment of urban green infrastructure in Armenia, focusing on three major cities, Yerevan, Gyumri, and Vanadzor, that represent contrasting climatic conditions. The study combines an analysis of the present climatic conditions and green‐space structure with a long‐term assessment of dendrological dynamics over the past 65 years (1960–2025) and an evaluation of potential changes in the species composition under projected climate‐change scenarios through 2100. Promising families and genera for arid and semiarid urban landscaping were identified, including Cupressaceae (*Juniperus*), Aceraceae (*Acer*), Caprifoliaceae (*Lonicera*, *Viburnum*, *Sambucus*), Fabaceae (*Albizzia*, *Caragana*, *Cercis*, *Laburnum*, *Sophora*), and Rosaceae (*Cotoneaster*, *Chaenomeles*, *Spiraea*, *Crataegus*, *Padus*, *Pyracantha*, *Rosa*, *Sorbus*), which exhibit the highest dendrological diversity. Approximately 70 ornamental trees and shrubs are recommended for use in urban green spaces, with key information provided on their ecological adaptability, tolerance to urban environmental conditions, and climatic requirements. Plantings of these species in urban and suburban areas can support multiple ecosystem functions, including recreational, hygienic, ecological, social, and cultural services. Overall, the findings provide a scientifically grounded basis for climate‐resilient green infrastructure planning, with relevance for semiarid and arid regions of Armenia, the Caucasus, and other regions with comparable environmental settings.

## 1. Introduction

Green construction is one of the important and mandatory elements of modern urban planning and forestry. Beautiful green plantations and forest areas regulate the surrounding microclimate and sanitary conditions, creating a favorable environment for people’s recreation. Construction, landscaping, and reforestation work must be carried out jointly.

In the conditions of constantly growing urbanization, the modern city faces many ecological problems. Currently, the cities and towns of the Caucasus are experiencing intensive construction and high population density. In Armenia, about 63% of the population now lives in urban areas. In the capital, Yerevan, as well as in other large cities, there is a pressing need to reconstruct and optimize public green spaces and forested areas by using highly decorative and environmentally sustainable plant species. Green plantations per resident of Yerevan are about 8 m^2^, which is below the minimum standard adopted by the World Health Organization, which is 9 m^2^ per capita. Green spaces and forest woody plants release oxygen, purify the air, absorb carbon dioxide and pollutant emissions, mitigate the impacts of global climate change, offer shade, reduce rainwater runoff, dampen traffic noise, and contribute a natural aesthetic to urban landscapes [1–14].

Management and development of urban green infrastructure, including the selection of plant species and sound reforestation and afforestation, care for them, and regular irrigation of seedlings, should be carried out taking into account the current and forecasted problems of climate change. Research exploring the nexus between green infrastructure, human health outcomes, and climate change assumes importance due to its potential in aiding cities, towns, and villages in climate change adaptation [6–8, 15–28]. The appropriate selection of dendrological composition, incorporating representatives of native flora alongside introduced species, and the systematic assessment of the ecological condition of urban green spaces are increasingly recognized as essential components of sustainable urban development [29–32].

While urban green infrastructure has been examined in various regions and addressed in a number of studies in Armenia over the past decades, comprehensive, integrative, city‐scale assessments that simultaneously consider species composition, functional characteristics, and climate‐change adaptation potential remain limited. In this context, this study aims to conduct a comparative assessment of the current state of green infrastructure in major Armenian cities, Yerevan, Gyumri, and Vanadzor, characterized by contrasting climatic conditions, through a systematic analysis of tree species composition, and their ecological and functional characteristics, as well as their adaptive potential to projected climate‐change scenarios.

## 2. Materials and Methods

### 2.1. Study Area

**FIGURE 1:**
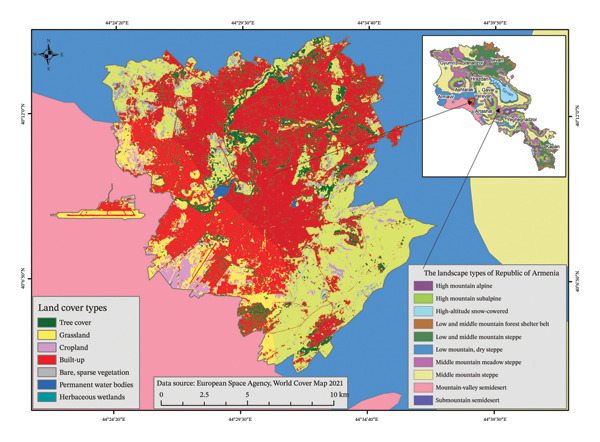
Land use of Yerevan city in relation to the landscape zones of the Republic of Armenia.

Study was conducted in urban green spaces of three major cities of Armenia: Yerevan, Gyumri, and Vanadzor. Yerevan is situated at an altitude of 900 to 1,350 m above sea level in the central part of Armenia. The city spans two major natural landscape zones: mountain‐valley semidesert and submountain semidesert (Figure 1). The climate of Yerevan is arid, characterized by extremely hot, dry, and long summers, as well as cold winters with occasional frosts and rare snowfall. The absolute minimum temperature in winter can drop to −27°C, while the maximum in summer can reach 41.2°C. Average annual precipitation ranges from approximately 340 mm/year in the low‐lying areas to 500 mm/year in the upper zones [33].

**FIGURE 2:**
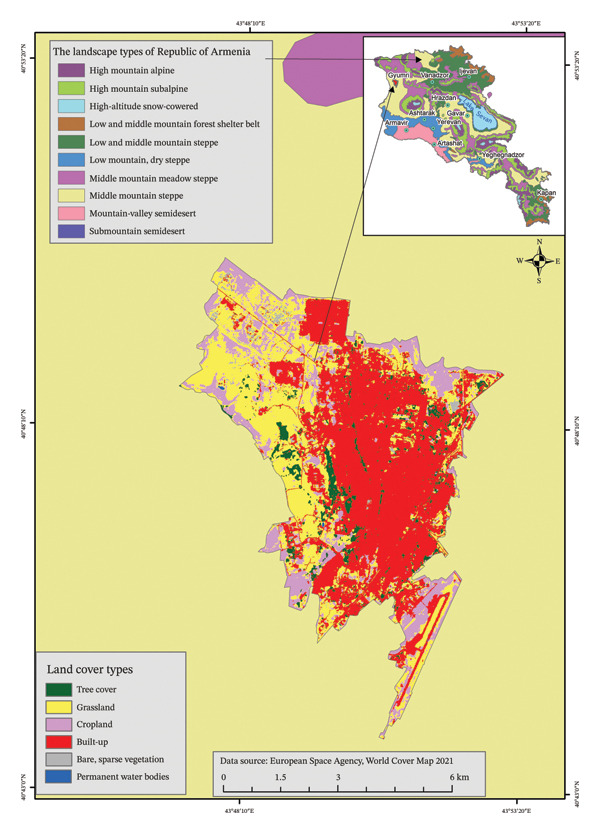
Land use of Gyumri city in relation to the landscape zones of the Republic of Armenia.

Gyumri is located at an altitude of 1,550–1,580 m above sea level in the northwestern part of the country. The city is entirely situated within the mountain steppe zone (Figure 2).

The climate is mountainous and arid, characterized by relatively mild summers and cold winters. The average annual air temperature in Gyumri is +6.2°C. Summers are warm, with August being the hottest month, when the average temperature reaches +19°C and the maximum can rise to +35°C. Winters are cold and snowy; the average temperature in January is −10°C, with minimum temperatures dropping to −35°C. The average annual precipitation is approximately 500 mm/year.

**FIGURE 3:**
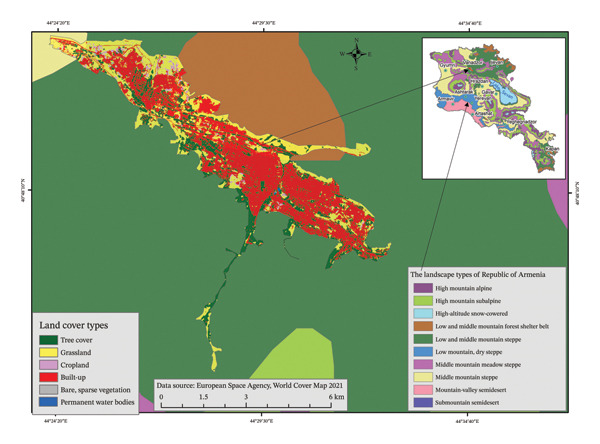
Land use of Vanadzor city in relation to the landscape zones of the Republic of Armenia.

The city of Vanadzor is located in northern‐central Armenia, on an average 1350 m altitude above sea level. It is mostly located in the mountain forest landscape zone (Figure 3). The city has a moderately mild, relatively humid climate. The average annual temperature is +7.3°С, the average temperature in January is −4.2°С, the average temperature in July is +19.7°С; the absolute maximum is +36°С, and the absolute minimum is −32°С. The average annual precipitation is 650 mm/year. The maximum amount of precipitation is observed in May–June.

### 2.2. Inventory of Woody Plants and Sampling Strategy

The study focused on assessing the species composition of urban woody vegetation, evaluating key ecological and biological indicators, identifying sustainable tree and shrub species, and analyzing green‐space coverage and plantation availability per capita. An inventory of tree and shrub species used in urban landscaping was conducted using a continuous route survey combined with selective recording. Field surveys were carried out within representative urban green spaces. Woody plants were recorded continuously along survey routes, with individual trees and shrubs treated as observation units. Surveys were conducted during the vegetation period (April–September/October) from 2018 to 2025. To ensure reliable species identification and accurate assessment of plant condition, key sites were revisited five to six times per growing season.

A continuous route survey combined with selective recording was applied to assess dendrological diversity in urban and suburban green spaces. Survey routes encompassed streets, residential areas, public parks, institutional territories, and special‐purpose sites, following the spatial distribution of green infrastructure across major administrative districts. In each city, six to eight routes were established, with a total length of approximately 12–18 km per city. Individual trees and shrubs, including linear street plantings, served as observation units, and all individuals encountered along the routes were recorded. For green spaces with clearly defined boundaries (e.g., public parks), spatial coverage was quantified as the cumulative surveyed area based on official documentation and field verification. The total area of urban green spaces differed in three cities and is approximately 860 hа in Yerevan, 270 ha in Gyumri, and 80–120 ha in Vanadzor, including parks, lawns, and street plants. Linear or fragmented green elements (e.g., street plantings, courtyards, buffer zones) were quantified by route length rather than area. Surveyed areas were selected to include all major functional types of urban green infrastructure, represent spatial distribution across districts and reflect prevailing species composition, planting structure, and management intensity. Overall spatial coverage was characterized by a combination of the cumulative area of defined green objects and the total length of survey routes.

### 2.3. Species Suitability Assessment

Species suitability scores were assigned based on three integrated criteria, drawing on field observations (2018–2023), expert evaluations, and literature data [29]: (1) survival and growth stability under urban conditions, (2) climatic and ecological tolerance (temperature, moisture, and light), and (3) resistance to urban stress and maintenance requirements. Species demonstrating consistently high survival, broad ecological tolerance, and low maintenance needs were classified as highly suitable (“++”) and recommended for mass planting. Species showing moderate performance or specific site limitations were classified as moderately suitable (“+”), while species with low survival or narrow ecological tolerance were considered unsuitable (“—”) for urban landscaping in the respective city or altitudinal zone. Suitability categories are summarized in Table 1.

**TABLE 1:** Characteristics of ornamental trees and shrubs recommended for use in urban plantations in Armenia.

Species	A life form	Ecological adaptability: attitude toward environmental factors			Suitability for towns			
		Temperature	Light	Humidity	Yerevan		Gyumri	Vanadzor
					Up to 1110 m	1100 m and more		
1	2	3	4	5	6	7	8	9
*Biota orientalis*	CT	HL, CR	ShL	DR	++	++	++	++
*Chamaecyparis lawsoniana*	CT	MCR, CR	ShL	ML	+	—	—	++
*Cupressus arizonica*	CT	MCR, CR	LL	МML	+	—	—	++
*Juniperus chinensis* “Variegata”	CT	CR	LL	DR	++	++	++	++
*J. communis* “Fastigiata”	CT	CR	LL	ML	+	++	+	++
*J. sabina*	CSh	CR	LL	МML	++	++	+	++
*J. virginiana*	CT	CR	ShL	МML	++	++	+	++
*Thuja occidentalis*	CT	CR	ShL	ML	++	++	++	++
*Ginkgo biloba*	DT	CR	LL	DR	+	+	—	+
*Abies nordmanniana*	CT	HL	ShL	ML	—	—	—	+
*Picea pungens* “Glauca”	CT	CR	LL	DR	++	++	++	++
*Pinus hamata*	CT	CR	LL	DR	++	++	+	++
*Cryptomeria japonica* “Elegans”	CT	MCR	ShL	МML	++	+	—	++
*Acer platanoides*	DT	CR	ShL	ML	+	++	+	++
*A. pseudoplatanus*	DT	CR	ShL	ML	+	++	+	++
*A. pseudoplatanus* “Purpureum”	DT	CR	LL	ML	+	++	+	++
*Berberis vulgaris* “Atropurpurea”	DSh	CR	LL	DR	++	++	+	++
*Betula litwinowii*	DT	CR	LL	МML	++	++	+	++
*Campsis radicans*	DL	CR	LL	ML	++	++	—	++
*Catalpa bignonioides*	DT	HL, CR	LL	МML	++	+	—	++
*Buxus balearica*	ESh	MCR	ShL	ML	+	++	—	++
*Lonicera flava*	DL	CR	ShL	ML	+	++	+	++
*L. maackii*	DSh	CR	ShL	DR	++	++	++	++
*Mahonia aquifolium*	ESh	CR	LL	DR	++	++	++	++
*Sambucus nigra*	DSh	MCR	ShL	ML	++	++	—	++
*Symphoricarpos albus*	DSh	CR	LL	DR	++	++	+	++
*Weigela floribunda*	DSh	CR	LL	ML	+	+	—	++
*Euonymus japonica*	ESh	HL	ShL	ML	+	—	—	++
*Corylus colurna* EN^∗^	DT	HL	ShL	ML	+	—	—	++
*Albizzia julibrissin*	DT	HL	LL	DR	++	—	—	++
*Caragana arborescens*	DSh	CR	ShL, LL	DR	++	++	++	++
*C. arborescens* “pendula”	DSh	CR	LL	DR	++	++	+	++
*Cercis griffithii* CR^∗^	DT	HL	LL	DR	++	+	—	++
*C. canadensis*	DT	CR	LL	DR	++	++	—	++
*Laburnum anagyroides*	DT	CR	ShL	ML	+	—	—	++
*Sophora japonica*	DT	HL, CR	ShL	DR	++	+	—	++
*Wisteria sinensis*	DL	HL, CR	LL	ML	++	++	—	++
*Quercus robur*	DT	CR	LL	DR	++	++	++	++
*Aesculus hippocastanum*	DT	CR	ShL	ML	+	++	—	++
*Deutzia scabra*	DSh	MCR	LL	DR	++	+	—	++
*Philadelphus caucasicus*	DSh	HL, CR	LL	DR	++	++	+	++
*Buddleia davidii*	DSh	HL	LL	DR	++	—	—	++
*Liriodendron tulipiferum*	DT	HL	LL	ML	—	—	—	+
*Magnolia liliiflora*	DT	HL	LL	ML	++	+	—	++
*Hibiscus syriacus*	DSh	HL, MCR	LL	DR	++	++	—	++
*Forsythia intermedia*	DSh	CR	LL	ML	++	++	+	++
*Fraxinus pennsylvanica*	DT	CR	LL	ML	++	++	++	++
*Ligustrum vulgare*	DSh	CR	LL, ShL	DR	++	++	++	++
*Platanus acerifolia*	DT	CR	LL	МML	++	++	+	++
*P. orientalis* EN^∗^	DT	MCR	LL	МML	++	+	—	++
*Chaenomeles japonica*	DSh	HL, CR	LL	DR	++	++	++	++
*Cotoneaster horizontalis*	DSh	CR	LL	DR	++	++	++	++
*Crataegus macracantha*	DT	CR	LL	МML	++	++	+	++
*Padus avium*	DT	CR	ShL	ML	+	++	+	++
*Pyracantha coccinea*	DSh	CR	LL	DR	++	++	++	++
*Rosa hemisphaerica*	DSh	CR	LL	DR	++	++	++	++
*Sambucus nigra* “Laciniata”	DSh	CR	ShL	ML	+	++	+	++
*Sorbus aucuparia*	DT	CR	ShL	DR	++	++	++	++
*Spiraea japonica*	DSh	CR	LL	МML	++	++	—	++
*Populus bolleana*	DT	CR	LL	ML	++	++	+	++
*Koelreuteria paniculata*	DT	HL	LL	DR	++	++	—	++
*Staphylea pinnata* VU^∗^	DSh	HL	ShL	ML	+	—	—	++
*Tilia caucasica*	DT	HL, CR	ShL	ML	+	+	—	++
*T. cordata*	DT	CR	ShL	ML	+	+	+	++
*Parthenocissus quinquefolia*	DL	CR	ShL	DR	++	++	+	++
*V. opulus* “Roseum”	DSh	CR	ShL	ML	++	++	+	++
*Vitis amurensis*	DL	CR	LL	МML	+	++	+	++

*Note:* Column 2: CT, coniferous tree; DT, deciduous tree; CSh, coniferous shrub; DSh, deciduous shrub; ESh, evergreen shrub; DL, deciduous liana. Column 3: HL, heat‐loving; MCR, medium cold‐resistant; CR, cold‐resistant. Column 4: ShL, shade‐loving; LL, light‐loving. Column 5: ML, moisture‐loving; МML, medium moisture‐loving; DR, drought‐resistant. Column 6–9: “++,” can grow massively; “+,” can grow moderately; “—,” cannot grow. Suitability categories were defined as follows: “++” indicates the species demonstrating high survival (> 70%), stable growth, and low maintenance demands, suitable for mass planting; “+” indicates the moderate performance with certain ecological or management limitations; “—” indicates the poor survival or ecological mismatch, making the species unsuitable for regular urban use.

^∗^Species, listed in *The Red Book of Plants of the Republic of Armenia* [34].

### 2.4. Plant Condition and Drought Resistance Assessment

The condition of green spaces in the cities of Yerevan, Gyumri, and Vanadzor was examined using the expedition‐route method. The condition of woody plants was assessed visually according to the methodology of Nikolaevsky et al. [35], based on crown damage, number of living branches, degree of foliage or needle retention, leaf damage, growth suppression, reduction in biometric parameters, and presence of diseases. Drought resistance of tree and shrub species was evaluated using visual assessment and standardized criteria described by Kuzmichev and Ovcharenko [36] and Belyuchenko and Mustafaev [37].

### 2.5. Species Identification and Reference Materials

Taxonomic identification and verification of tree and shrub species were carried out using herbarium materials from the Herbarium (ERE) of the A. Takhtajan Institute of Botany (NAS RA) and relevant dendrological literature, including *Dendroflora of the Caucasus* [38], Sosnowsky and Makhatadze [39], Vardanyan [40], and the *Annotated Catalog of Trees and Shrubs of Botanical Gardens and Arboretums of Armenia* [41].

### 2.6. Data Analysis

Taxonomic diversity of urban green spaces was assessed using the Shannon diversity index (*H* ^′^) [42, 43], calculated as

(1) H′=−∑i=1npilnpi,

where *p*_*i*_ is the proportion of species assigned to the *i*th plant family and *n* is the total number of families. Here, *p*_*i*_ was calculated as the number of species in the *i*th family (*S*_*i*_) divided by the total number of recorded species (∑*S*). Although the Shannon index is traditionally applied at the species level, its use at higher taxonomic ranks allows the assessment of taxonomic diversity and its temporal dynamics given the structure of the available datasets.

Climatic trends in annual air temperature and precipitation for the studied cities were analyzed using long‐term meteorological data. The Mann–Kendall test was applied to detect statistically significant monotonic trends, and Sen’s slope estimator was used to quantify the rate of change.

### 2.7. Ethical Considerations and Permissions

All field surveys were noninvasive and conducted exclusively in publicly accessible urban areas. In accordance with local regulations, formal municipal permits were not required. Nonetheless, the relevant municipal authorities (Yerevan, Gyumri, and Vanadzor Municipalities) were officially informed about the research activities, ensuring transparency and institutional awareness of the study. Protected plant species listed in the Red Book of Plants of the Republic of Armenia (*Corylus colurna*, *Cercis griffithii*, *Platanus orientalis*, and *Staphylea pinnata*), recommended for landscaping, were propagated exclusively in authorized nurseries and were not collected from wild populations; all activities fully complied with national conservation and ethical requirements.

## 3. Results

Yerevan is a rapidly developing urban center. Accompanying this growth is a substantial increase in industrial activities, vehicular traffic, and the extent of paved and asphalted surfaces. Such intensified urbanization adversely affects public health and contributes to the degradation of climatic conditions, air and water quality, and soil properties. Green spaces and urban forests play a critical role in mitigating these negative environmental impacts and enhancing urban ecosystem resilience.

The expansion of plantations and the enrichment of the dendrological composition with highly ornamental tree species, however, are severely constrained by the harsh semidesert climatic conditions, which include hot, nearly precipitation‐free summers and frequently frosty winters with little or no snow cover, as well as soils of low fertility.

**FIGURE 4:**
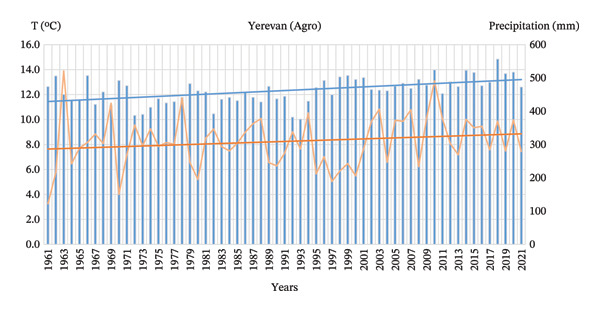
Long‐term trends in mean annual air temperature and total annual precipitation at the Yerevan Agro Meteorological Station (1961–2021).

In terms of climate change, the mean annual air temperature at the Yerevan Agro Meteorological Station has increased by more than 1.5°C during 1961–2021 and exhibits a clear and persistent upward trend. The consistency of the trend throughout the observation period suggests a robust signal of regional warming. Annual precipitation is characterized by strong interannual variability and remains generally stable over the observation period, without a pronounced long‐term trend (Figure 4).

Analysis of annual temperatures in Yerevan demonstrates a pronounced warming trend. The Mann–Kendall test yielded a *Z* score of 4.27, exceeding the critical threshold for the 99% confidence interval, indicating a highly significant monotonic increase. According to Sen’s slope estimator, the warming rate is approximately 0.0313°C per year, or roughly 0.31°C per decade. This rate surpasses the global land average, highlighting a clear regional amplification of warming. The trendline reflects a steady rise in temperatures, signaling a consistent and accelerated warming pattern in the city.

In contrast, total annual precipitation in Yerevan shows no statistically significant long‐term trend. The Mann–Kendall test produced a *Z* score of 1.27 with a *p* value of 0.204, while Sen’s slope is small and not meaningful in the context of natural variability. Interannual fluctuations are pronounced, with extreme highs (e.g., 521 mm in 1963) and lows (122 mm in 1961), illustrating a highly variable and volatile precipitation regime. These fluctuations make long‐term water management more challenging, even in the absence of a clear directional trend.

Overall, Yerevan is experiencing a distinct warming phase, while precipitation totals remain relatively stable over the long term. The increase in temperature likely enhances evaporation rates, potentially leading to drier soils and greater stress on water resources, despite unchanged precipitation volumes. This “New Normal” for Yerevan’s climate is characterized by higher mean temperatures and more frequent extreme thermal events, emphasizing the need for adaptive urban planning and sustainable water management strategies.

Observations have shown that early spring and late autumn frosts characteristic of the local climate act as limiting factors for plant growth and development. Consequently, over time, several tree species insufficiently adapted to local ecological conditions, including *Cedrus deodara*, *Metasequoia glyptostroboides*, *Cupressus sempervirens* “*Pyramidalis*,” and *Parrotia persica*, have been partially damaged or have declined. In the lowland microclimatic zones of the city, at elevations up to 1000 m (Shengavit, Erebuni, Malatia‐Sebastia, and the city center), species such as *Albizia julibrissin*, *Euonymus japonicus*, *Ced. deodara*, and *Larix leptolepis* are found, whereas they are absent from higher districts located at 1200–1300 m (Avan, Kanaker‐Zeytun, and Nor‐Nork) due to their low frost resistance.

Yerevan, one of the largest cities in the region, has a population exceeding one million inhabitants. According to established urban planning standards, the provision of green space should be 10 m^2^ per resident citywide, 14 m^2^ in residential areas, and 24 m^2^ for public use. However, current measurements indicate that the green area per resident is only 7.8 m^2^.

An inventory of urban plantations across different types and categories, including central streets and avenues, revealed that the most commonly planted tree species include *P. orientalis*, *Fraxinus excelsior*, *F. pennsylvanica*, *Quercus robur*, *Populus bolleana*, *Robinia pseudoacacia*, *R. pseudoacacia* “*Compacta*,” *Sophora japonica*, *Thuja occidentalis*, *Biota orientalis*, and *Juniperus virginiana*. Among ornamental shrubs, the most frequently utilized species were *Spiraea* × *vanhouttei*, *Ligustrum vulgare*, *Philadelphus caucasicus*, *Forsythia intermedia*, *Chaenomeles japonica*, *Syringa vulgaris*, *Berberis vulgaris*, *Sambucus nigra*, *Buxus sempervirens*, and *Cornus alba*. These results provide a comprehensive overview of the species composition in Yerevan’s urban green infrastructure and highlight the predominance of both native and widely adapted ornamental species.

An analysis of the dynamics of the taxonomic composition of trees and shrubs in Yerevan over 65 years revealed a decrease in the number of species from 186 to 149, while the number of genera remained relatively stable (72 genera in 2018–2025) (Table 2). The reduction primarily is related to the energy crisis of the 1990s, when, on the one hand, urban green spaces were cut down without hindrance, and on the other hand, trees were not cared for and watered. As a result, the number of three to five species from the number of families, such as Caprifoliaceae, Fabaceae, Fagaceae, Oleaceae, and Rosaceae, was reduced. Currently, the introduced plants occupy a predominant place in the greening and лесных массивов of Yerevan. Aboriginal flora includes the species of *Ac. campestre*, *Ac. platanoides*, *B. vulgaris*, *Betula litwinowii*, *Carpinus betulus*, *Cerasus mahaleb*, *Clematis orientalis*, *C. avellana*, *Cotinus coggygria*, *F. excelsior*, *Padus racemosa*, and *Ph. caucasicus*. Recent dendrological analyses covering 2018–2025 demonstrate a recovery in diversity, with approximately 170 species representing 39 families and 83 genera incorporated into Yerevan’s urban landscaping. A substantial proportion of the tree and shrub species used belonged to two major families, Rosaceae and Fabaceae, each represented by 10 or more genera. The families Oleaceae, Pinaceae, Caprifoliaceae, Moraceae, Fagaceae, and several others also exhibited relatively high taxonomic diversity, comprising three to five genera.

**TABLE 2:** Dynamics of the number of genera and species of trees and shrubs in Yerevan green spaces over 65 years (1960–2018/25) based on historical and recent investigations.

Taxonomic composition of trees and shrubs in the green spaces of Yerevan						
Family	1960		1985		2018–2025	
	Genera	Species	Genera	Species	Genera	Species
*Aceraceae*	1	9	1	9	1	8
*Anacardiaceae*	2	2	2	2	1	1
*Berberidaceae*	2	5	2	2	2	3
*Betulaceae*	3	4	3	4	3	6
*Bignoniaceae*	2	4	2	3	2	3
*Buxaceae*	1	2	1	1	1	2
*Caprifoliaceae*	4	10	1	2	3	7
*Celastraceae*	1	3	1	1	1	2
*Cornaceae*	1	3	1	2	1	3
*Cupressaceae*	3	8	3	6	3	10
*Elaeagnaceae*	2	2	2	2	2	2
*Fabaceae*	12	16	7	9	9	13
*Fagaceae*	3	7	2	4	1	5
*Ginkgoaceae*	1	1	—	—	1	1
*Grossulariaceae*	2	3	1	2	1	3
*Hydrangeaceae*	2	8	2	3	2	4
*Moraceae*	4	6	3	6	4	6
*Oleaceae*	5	15	4	13	4	10
*Pinaceae*	5	11	4	6	3	6
*Platanaceae*	1	3	1	3	1	3
*Rhamnaceae*	2	2	—	—	2	3
*Rosaceae*	19	31	15	25	16	27
*Salicaceae*	2	15	2	12	2	10
*Sapindaceae*	1	1	—	—	1	1
*Tamaricaceae*	1	1	1	1	1	1
*Tiliaceae*	1	4	1	4	1	2
*Ulmaceae*	2	8	1	6	1	5
*Vitaceae*	2	2	2	2	2	2
**In total**	**87**	**186**	**65**	**130**	**72**	**149**

*Note:* Historical data were obtained from the studies of Harutyunyan [34] and the *Annotated Catalog of Trees and Shrubs of Botanical Gardens and Arboretums of Armenia* [41].

In the town of Gyumri, the total length of street plantings reaches 15 km. Parks and gardens established over the past decade are generally in good condition. The total area occupied by green plantations is approximately 150 ha, of which 144.4 ha is covered by gardens, parks, and groves, while sown areas of flowers and lawns account for 6.0 ha. An analysis of the dynamics of the taxonomic composition of trees and shrubs in Yerevan over 65 years revealed a decrease in the number of species from 186 to 149, while the number of genera remained relatively stable (72 genera in 2018–2025) (Table 2). The reduction primarily is related to the energy crisis of the 1990s, when, on the one hand, urban green spaces were cut down without hindrance, and on the other hand, trees were not cared for and watered. As a result, the number of three to five species from the number of families, such as Caprifoliaceae, Fabaceae, Fagaceae, Oleaceae, and Rosaceae, was reduced. Currently, the introduced plants occupy a predominant place in the greening and лесных массивов of Yerevan. Aboriginal flora includes the species of *Ac. campestre*, *Ac. platanoides*, *B. vulgaris*, *Be. litwinowii*, *Ca. betulus*, *Cerasus mahaleb*, *Cl. orientalis*, *C. avellana*, *Co. coggygria*, *F. excelsior*, *Pa. racemosa*, and *Ph. caucasicus*. Recent dendrological analyses covering 2018–2025 demonstrate a recovery in diversity, with approximately 170 species representing 39 families and 83 genera incorporated into Yerevan’s urban landscaping. A substantial proportion of the tree and shrub species used belonged to two major families, Rosaceae and Fabaceae, each represented by 10 or more genera. The families Oleaceae, Pinaceae, Caprifoliaceae, Moraceae, Fagaceae, and several others also exhibited relatively high taxonomic diversity, comprising three to five genera.

In the town of Gyumri, the total length of street plantings reaches 15 km. Parks and gardens established over the past decade are generally in good condition. The total area occupied by green plantations is approximately 150 ha, of which 144.4 ha are covered by gardens, parks, and groves, while sown areas of flowers and lawns account for 6.0 ha.

**FIGURE 5:**
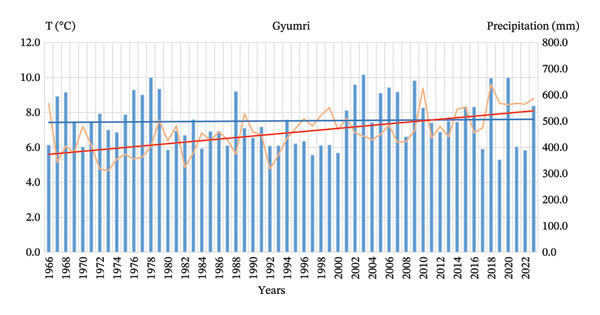
Trends in mean annual air temperature and total annual precipitation in Gyumri for the period 1966–2023.

The area of green space per capita is approximately 13.4 m^2^. In terms of climate change, the mean annual air temperature in Gyumri increased by more than 2.4°C over the period 1966–2023. The temperature trend is monotonic and statistically robust, indicating an intensified warming signal in the northern part of Armenia. Annual precipitation in Gyumri exhibits considerable year‐to‐year variability, with no statistically significant long‐term trend detected (Figure 5). Low winter temperatures, short vegetation, lack of moisture, and strong winds are considered limiting factors for the growth and development of trees.

Analysis of the annual mean temperature in Gyumri over the period 1966–2023 reveals a statistically significant upward trend. The Mann–Kendall test yielded *S* = 747, *Z* = 5.00, and *p* = 5.6 × 10^−7^, indicating strong evidence against the null hypothesis of no trend. Sen’s slope estimates of +0.0469°C/year correspond to an increase of approximately 0.47°C per decade. These results demonstrate a robust warming signal in Gyumri during the study period.

Analysis of annual total precipitation over the same period shows no statistically significant trend. The Mann–Kendall test returned *S* = 19, *Z* = 0.12, and *p* = 0.904. Although Sen’s slope is slightly positive (+0.17 mm/year, ≈1.7 mm/decade), this change is negligible and falls within the range of random variability. Therefore, no meaningful trend in precipitation can be inferred.

Overall, the analysis points to a clear warming trend in Gyumri, while precipitation patterns remain highly variable without a significant directional change. This combination of warming temperatures and stable precipitation has important implications for agriculture, water resource management, and local climate adaptation planning in the Shirak region.

An inventory of urban green spaces revealed that the most commonly planted tree and shrub species include *Pi. hamata*, *J. virginiana*, *Thuja occidentalis*, *Ac. campestre*, *Ac. negundo*, *F. excelsior*, *P. alba*, *P. nigra*, *P. simonii*, *R. pseudoacacia*, *Ulmus laevis*, *Caragana arborescens*, *Lonicera tatarica*, and *Ribes nigrum*, among others. These data provide a comprehensive overview of the species composition of Gyumri’s urban plantations and indicate the predominance of both native and widely adapted ornamental species.

Arboricultural research has been conducted in the town of Gyumri since the 1960s. Approximately 120 species belonging to 31 families and 67 genera were present in the city’s green spaces. Comparative analysis of our studies (2018–2025) indicates that more than 60 tree and shrub species, representing 16 families and 31 genera, are currently used in the landscaping of the city (Table 3). Most of the trees and shrubs now growing in Gyumri belong to the following families: Rosaceae (8 genera, 10 species), Salicaceae (2 genera, 11 species), Oleaceae (3 genera, 7 species), and Pinaceae (2 genera, 6 species). Among the native dendroflora incorporated into the city’s landscaping are *F. excelsior*, *Pi. hamata*, *Ac. campestre*, *Ac. platanoides*, and *Ri. reclinatum*, among some others.

**TABLE 3:** Dynamics of the number of genera and species of trees and shrubs in Gyumri green spaces over 65 years (1960–2018/25) based on historical and recent investigations.

Taxonomic composition of trees and shrubs in the green spaces of Gyumri						
Family	1960		1985		2018–2025	
	Genus	Species	Genus	Species	Genus	Species
Pinaceae	3	8	2	2	2	6
Cupressaceae	3	4	1	1	2	3
Aceraceae	1	6	1	4	1	4
Hippocastanaceae	1	1	1	1	1	1
Fabaceae	8	11	2	2	2	3
Rosaceae	14	23	8	10	8	10
Fagaceae	2	3	—	—	1	1
Ulmaceae	2	6	1	2	1	4
Oleaceae	4	9	3	4	3	7
Grossulariaceae	2	3	1	1	2	3
Juglandaceae	1	2	1	1	1	1
Caprifoliaceae	3	8	2	2	2	3
Hydrangeaceae	1	1	—	—	1	1
Salicaceae	2	11	2	9	2	11
Anacardiaceae	1	1	—	—	1	1
Tiliaceae	1	2	1	1	1	1
**In total**	**49**	**99**	**26**	**40**	**31**	**60**

*Note:* Historical data were obtained from the studies of Harutyunyan [44].

**FIGURE 6:**
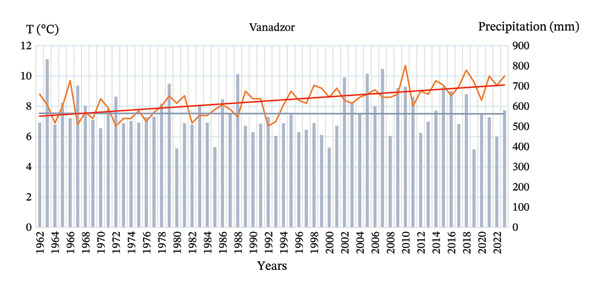
Long‐term variations in mean annual air temperature and total annual precipitation in Vanadzor during 1962–2023.

The climatic conditions of the city Vanadzor are sufficiently favorable for the growth and development of plants of different geographical origins. In terms of climate change, in Vanadzor, the mean annual air temperature rose by more than 2.1°C during 1962–2023 and shows a pronounced upward tendency, consistent with observed warming patterns across the region; a weak decreasing tendency in annual precipitation is observed in Vanadzor, although interannual variability remains high (Figure 6).

Analysis of annual temperatures in Vanadzor from 1962 to 2023 reveals a clear upward trend. The Mann–Kendall test produced a *Z* score of 5.52 and a *p* value of 0.001, indicating statistical significance at the 95% confidence level. Sen’s slope estimator indicates a warming rate of approximately 0.0368°C per year, amounting to an overall increase of roughly 2.25°C over the study period. The trendline shows a pronounced rise, particularly noticeable from the early 1990s onward, highlighting a consistent warming signal in the region.

In contrast, annual precipitation in Vanadzor does not exhibit a significant long‐term trend. The Mann–Kendall test returned a *Z* score of −0.22 and a *p* value of 0.82218, confirming the absence of a statistically meaningful change. Sen’s slope is slightly negative (−0.1026 mm per year), but this minor change falls within the natural interannual variability, which ranges from approximately 380 mm to over 800 mm. Such fluctuations mask any persistent trends, suggesting that the regional water cycle remains relatively stable despite variations from year to year.

Overall, the analysis indicates that Vanadzor is experiencing a distinct warming trend, consistent with broader regional climate‐change observations. While temperatures show a steady and significant increase, precipitation patterns remain highly variable without a clear directional trend. These findings have important implications for urban planning, agricultural water management, and assessing the local environmental impacts of a warming climate in the Lori region.

In the town of Vanadzor, the main tree species found in street plantings and gardens include *F. excelsior*, *Ac. pseudoplatanus*, *Ac. campestre*, *Aesculus hippocastanum*, *Tilia caucasica*, *P. gracilis*, *Thuja occidentalis* “*Columnaris*,” *B. orientalis*, and *Picea abies*. Common shrub species include *Li. vulgare*, *Buxus sempervirens*, and *Spiraea* × *vanhouttei*, while *L. sibirica*, *Ca. orientalis*, *Be. pendula*, and *Sorbus persica* are rarely found in gardens. The area of tree plantings per capita in Vanadzor is approximately 14.26 m^2^.

Targeted studies on the state of green spaces in Vanadzor have not been conducted previously. However, our research indicates that the species composition has not undergone major changes, as most trees in the town’s green spaces are part of older plantings. Currently, 30 species from 12 families and 19 genera are present in these areas, with the families Pinaceae and Aceraceae being relatively well represented (Table 4).

**TABLE 4:** Dynamics of the number of genera and species of trees and shrubs in Vanadzor green spaces in 1985–2018/25 based on historical and recent investigations.

Taxonomic composition of trees and shrubs in the green spaces of Gyumri				
Family	1985		2018–2025	
	Genus	Species	Genus	Species
Pinaceae	3	5	3	5
Cupressaceae	2	3	2	3
Aceraceae	1	4	1	5
Hippocastanaceae	1	1	1	1
Rosaceae	5	6	3	4
Betulaceae	1	1	1	2
Buxaceae	1	1	1	1
Fagaceae	1	1	1	1
Ulmaceae	1	2	1	2
Oleaceae	2	2	2	2
Salicaceae	2	4	2	2
Tiliaceae	1	2	1	2
**In total**	**21**	**32**	**19**	**30**

*Note*: Historical data were obtained according to *Annotated catalog* … [41].

The taxonomic diversity of urban green spaces in the studied cities was quantitatively assessed using the Shannon diversity index (*H* ^′^), calculated based on the proportional distribution of species among plant families. Species occurrence scores from historical (1960, 1985) and recent (2018–2025) surveys (Tables 2–4) and the detailed species assessment, including ecological adaptability and suitability for urban planting (Table 1), were used for this analysis. In Yerevan, *H* ^′^ was 3.05, reflecting relatively high dendrological diversity despite historical urban development pressures. Gyumri exhibited lower diversity, with *H* ^′^ = 2.65, indicating a more limited range of species and families in urban plantings. Vanadzor maintained intermediate and relatively stable diversity, with *H* ^′^ = 2.85, consistent with favorable climatic conditions and historically stable landscaping practices. These values corroborate the temporal patterns observed in historical and recent surveys, confirming that Yerevan and Gyumri experienced mid‐20th century declines in dendrological diversity with partial recovery by 2018–2025, whereas Vanadzor’s urban vegetation remained consistent throughout the studied period.

Across the studied cities, a statistically significant warming trend was consistently observed, while annual precipitation showed high interannual variability and no significant long‐term trend. Temperature increases were associated with changes in the taxonomic composition and structure of urban vegetation. In Yerevan, the urban plantings were dominated by species with higher tolerance to heat and water deficit. In Gyumri, rising temperatures coincided with more severe climatic limitations, resulting in a narrower range of tree and shrub species suitable for urban landscaping. Vanadzor exhibited a relatively stable dendrological composition. Variations in the Shannon diversity index reflected these patterns, with lower diversity observed in cities experiencing more pronounced climatic stress.

The Fourth National Climate Change Report [33] presents climate projections for Armenia up to 2100 under the RCP8.5 scenario using the METRAS model. Mean air temperature in Yerevan and the other studied cities is projected to increase annually and across all seasons, whereas precipitation changes are more variable. Total annual precipitation is expected to decrease overall, despite a slight increase in autumn precipitation and a more pronounced decline in the other seasons. Observations show a consistent rise in mean annual air temperature across all locations, while annual precipitation exhibits weak and spatially heterogeneous trends. Overall, climate projections indicate continued warming accompanied by a moderate reduction in precipitation toward the end of the 21st century (Table 5). Projected values represent the scenario‐based estimates derived from climate model outputs.

**TABLE 5:** Projected changes in mean annual air temperature and annual precipitation in Yerevan, Gyumri and Vanadzor relative to the 1961–1990 baseline.

City and towns	Temperature (°C)				Precipitation (mm)			
	Basic data of 1961–1990	2040	2070	2100	Basic data of 1961–1990	2040	2070	2100
Yerevan	10.8	12.4	14.1	15.5	343	332	322	313
Gyumri	5.5	7.1	8.8	10.2	502	486	472	458
Vanadzor	8.4	10.0	11.7	13.1	592	573	557	540

Climate‐change factors should be taken into account when selecting suitable tree species for landscaping in the cities under study. For this purpose, the examples based on these urban areas illustrate the forecasted temperature parameters for the medium and long terms. It is important to note that climate change is currently occurring at an accelerated pace, and its effects are observable worldwide, including in the Caucasus. Warming is accompanied by a potential deficit in precipitation, which is expected to increase in both the near and distant future. Forecast analyses provide essential information for bioecological management. In particular, consideration of both temperature and precipitation is critical for selecting plant species and varieties for cultivation in semidesert, submountain, and mountain urban landscapes that are exposed to multiple environmental stressors.

To enrich the species composition of trees and shrubs in urban green areas and to improve landscaping, particularly in Vanadzor and Gyumri, while also taking into account projected climate change, we have proposed a selection of decorative and valuable tree and shrub species suitable for the studied towns according to their functional purposes.

We conducted long‐term observations of plant growth and development in urban and suburban plantings, nurseries, and botanical gardens across different regions of Armenia during the vegetation seasons. These studies allowed us to identify overall plant condition, bioecological stability, and ornamental potential, based on a comprehensive set of morphological indicators for integrated ecological and sanitary assessments of trees and shrubs in both natural and cultivated conditions. The biological and ecological traits of plants were correlated with the weather, climatic, and geomorphological characteristics of the study areas.

In addition, we considered current research and scientific generalizations concerning the environmental tolerance and plasticity of woody plant species and varieties in response to limiting environmental factors.

The general information presented in Table 1 summarizes the species composition of the main trees suitable for use in the urban green spaces of Yerevan, Vanadzor, and Gyumri, along with their ecological characteristics, including responses to heat, light, and humidity, and their overall suitability for each city.

The plant species presented are promising for widespread introduction into cultivation to establish sustainable and highly ornamental plantings in urban, suburban, and rural areas. Ecosystems based on these species are capable of fulfilling territorial planning, ecological, aesthetic, social, and cultural functions. By incorporating the proposed species and varieties, it is possible to create resilient landscape‐salutogenic complexes. Consequently, these plants can also contribute to addressing issues of hygienic safety in populated areas and to promoting public health.

## 4. Discussion

The present study reveals clear differences in the species composition, taxonomic structure, and provision of urban green spaces among the three largest cities of Armenia: Yerevan, the capital of the country, and the major regional centers Gyumri and Vanadzor. These differences are primarily associated with climatic conditions, altitudinal gradients, and long‐term urban landscaping and maintenance practices.

In Yerevan, the predominance of introduced tree species, coupled with a declining share of native taxa, reflects a long‐standing emphasis on ornamental plantings in a semiarid climate. Our results indicate that this strategy has been associated with a decline in species richness and the loss of individual taxa in recent decades. Many introduced species exhibit limited tolerance to prolonged drought, high summer temperatures, and compacted urban soils, which reduces their stability and lifespan. Some exhibit satisfactory adaptation, while many species are partially adapted or fail to withstand the negative impacts of a number of factors [45]. Similar patterns have been observed in cities in arid and semiarid regions, where insufficient ecological plasticity limits the resilience of urban green spaces [46, 47].

Gyumri, located at higher altitudes and characterized by continental climatic conditions with rather harsh winters, maintains a more limited set of tree species. The decline in taxonomic diversity recorded in this city indicates the influence of limiting factors such as short vegetation periods and low winter temperatures. These limitations negatively impact plant viability and limit both the ornamental and ecological effectiveness of urban green spaces, a phenomenon also observed in other cities with cold climates and mountainous regions [48, 49]. Shannon diversity index (*H* ^′^) values calculated to assess the taxonomic diversity and ecological resilience of urban green spaces (Tables 1–4) showed that although Yerevan and Gyumri experienced a decline in tree and shrub diversity in the mid‐20th century, targeted introduction of drought‐tolerant and resilient species partially restored it by 2018–2025. In contrast, in Vanadzor with cool summers and relatively mild winters, diversity remained stable, reflecting the favorable climate and long‐standing planting practices.

The comparative analysis demonstrates that rising air temperature is a primary driver of changes in urban dendrological composition across different climatic zones of Armenia. Despite the absence of significant long‐term trends in precipitation, sustained warming contributes to increasing environmental stress, leading to selective retention of species with higher ecological adaptability. Similar patterns have been reported in other urbanized and semiarid regions. These findings support the necessity of climate‐adaptive species selection in urban landscaping under ongoing climate change. In semiarid cities, rising air temperatures associated with climate change and intensified by urban heat island effects act as a primary stressor for urban green spaces, driving species‐specific thermal responses, reducing the climatic suitability of temperature‐sensitive tree species, and ultimately leading to shifts in species composition and decreased resilience of urban vegetation [50–52].

Promising plant families and genera for urban landscaping in arid and semiarid regions have been identified, including Cupressaceae (*Juniperus*), Aceraceae (*Acer*), Caprifoliaceae (*Lonicera*, *Viburnum*, *Sambucus*), Fabaceae (*Albizzia*, *Caragana*, *Cercis*, *Laburnum*, *Sophora*), and Rosaceae (*Cotoneaster*, *Chaenomeles*, *Spiraea*, *Crataegus*, *Padus*, *Pyracantha*, *Rosa*, *Sorbus*), which together represent the highest dendrological diversity. A total of approximately 70 ornamental trees and shrubs are recommended for urban green spaces, selected based on their ecological adaptability, tolerance to urban environmental stressors, and specific climatic requirements. The study also highlighted gaps in the representation of native and ornamental woody species, including the near absence of native lianas in Gyumri and Vanadzor. Incorporating studied species contributes to the formation of resilient and functionally reliable urban phytocenoses, capable of maintaining vitality and performing key ecosystem services, including microclimate regulation, air purification, aesthetic enhancement, and social benefits, even under increasing climatic and anthropogenic pressures. In addition, due to their edificatory properties, these species perform important planning, ecological, and sanitary‐hygienic functions in urban ecosystems. Several native woody species used in urban landscaping, including *C. colurna*, *Ce. griffithii*, *P. orientalis*, and *S. pinnata*, are listed in the Red Book of Plants of the Republic of Armenia [53] and require protection in natural habitats. Their use in urban green spaces does not replace in situ conservation but may contribute to long‐term preservation by supporting ex situ and quasi in situ populations and reducing pressure on natural stands.

Observed climate trends in Armenia indicate a gradual increase in air temperature accompanied by a tendency toward reduced and more irregular precipitation, leading to progressive aridization and increased climatic variability. These changes have been documented in long‐term observational studies and national climate assessments [29, 33, 45]. The same tendencies are reflected in the climate projections used in this study (Table 5). These regional climatic changes were a key factor influencing the selection of tree and shrub species and varieties recommended for wider cultivation.

Tree and shrub species recommended in this study (Table 1) were selected based on long‐term field observations, ecological monitoring data, and their demonstrated performance under comparable environmental and urban conditions. The results emphasize the importance of diversifying urban species composition while prioritizing ecologically stable and functionally reliable taxa. Such an approach supports the formation of resilient urban phytocenoses capable of maintaining vitality and performing climatic regulation, sanitary‐hygienic, aesthetic, and social functions under increasing climatic and anthropogenic pressures [25, 54–56].

While urban green infrastructure has been previously examined in Armenia, existing studies have primarily focused on individual cities, specific plant groups, or isolated ecological indicators. The novelty of the present study lies in its integrated, city‐scale comparative assessment of green infrastructure across three major Armenian cities with contrasting climatic conditions. By simultaneously analyzing tree species composition, ecological and functional traits, and climate adaptation potential, this study provides a holistic framework for evaluating the resilience of urban green infrastructure under current and projected climate conditions. Importantly, the comparative approach allows the identification of common and city‐specific vulnerabilities and strengths, which have not been previously addressed in Armenian urban greening research.

Collectively, these findings indicate that, despite historical fluctuations, Yerevan’s urban plantings have maintained a relatively high level of dendrological diversity. Contemporary landscaping practices have not only preserved this diversity but also enhanced the ecological stability and aesthetic quality of the city’s green spaces, highlighting the ongoing importance of biodiversity‐informed urban planning. Overall, the findings confirm that scientifically grounded species selection, adaptive management practices, and continuous ecological monitoring are essential for improving the sustainability of urban and suburban green spaces in the Caucasus region under conditions of ongoing climate change. The patterns identified in this study are consistent with broader observations from climatically constrained regions and provide region‐specific evidence that can inform urban planning and sustainable greening strategies in Armenia and comparable environments of the Caucasus region.

Thus, the framework of this study aligns with international urban greening and climate adaptation approaches that emphasize resilience planning, ecosystem services provision, and climate‐responsive species selection. The comparative, long‐term dataset supports adaptation pathway thinking by identifying species with higher persistence under projected climatic stress. These findings may be transferable to other semiarid and mountainous cities facing similar thermal and moisture constraints, while accounting for local ecological and management contexts.

Although climate variables were the primary focus of this study, species performance in urban environments is also influenced by interacting local factors such as soil conditions, irrigation regimes, maintenance intensity, and urban heat island effects. Quantifying causal relationships between specific climatic thresholds and species decline or persistence would require dedicated physiological, soil, and microclimatic datasets that were beyond the scope of the present work. These interacting drivers represent the important directions for future research aimed at refining species suitability assessments under climate change.

## 5. Conclusion

This study provides the first integrated comparative assessment of urban green infrastructure in three major Armenian cities: Yerevan, Gyumri, and Vanadzor, linking species composition, functional characteristics, and climate adaptation potential. The results reveal both shared patterns and city‐specific challenges, indicating structural, ecological, and management‐related limitations that currently constrain the stability, vitality, and functionality of urban green spaces. An integrated analysis demonstrates that sustained regional warming, even in the absence of significant long‐term changes in precipitation, represents a key climatic factor shaping the structure and diversity of urban dendrological assemblages in the studied cities. Based on long‐term observations and ecological analyses, approximately 70 ornamental trees, shrubs, and lianas with sufficient adaptive potential were identified as suitable for urban landscaping under current and projected climate conditions. The implementation of this species assortment can enhance ecological functionality, environmental quality, aesthetic value, and resilience of urban green infrastructure.

Overall, the findings provide a scientifically grounded basis for climate‐resilient urban greening strategies, green infrastructure planning, and the sustainable development of urban ecosystems in Armenia, the Caucasus, and other regions with comparable environmental conditions.

## Funding

The work was done in the frames of the research project 21T‐1F140 “The eco‐biological aspects of optimization and phyto‐technical measures of urban plantation in the Republic of Armenia” supported by the Science Committee of the Ministry of Education, Science, Culture and Sports of the Republic of Armenia for which we express our gratitude.

## Conflicts of Interest

The authors declare no conflicts of interest.

## Data Availability

Raw data on species inventory, climate, and survey datasets used in this study are archived and available upon request from the Institute of Botany after A. Takhtajyan of the National Academy of Sciences of Armenia (official website: https://www.botany.sci.am/) and the Hydrometeorology and Monitoring Center SNCO of the Ministry of Environment of the Republic of Armenia (climate and meteorological data via https://env.am/en/environment/environmental-monitoring). Detailed field survey protocols, species matrices, and city‐level summary data supporting the results are likewise available from these repositories to facilitate replication and further analysis.
